# Recent Advances in Polymer Recycling: A Review of Chemical and Biological Processes for Sustainable Solutions

**DOI:** 10.3390/polym17050603

**Published:** 2025-02-24

**Authors:** Damián Peti, Jozef Dobránsky, Peter Michalík

**Affiliations:** Faculty of Manufacturing Technologies with a Seat in Presov, Technical University of Kosice, Štúrova St. 31, 080 01 Presov, Slovakia; damian.peti@tuke.sk (D.P.); peter.michalik@tuke.sk (P.M.)

**Keywords:** polymer composites, recycling, plastics, waste, production

## Abstract

Plastics, particularly synthetic organic polymers, have become indispensable in modern life, yet their large-scale production has led to significant environmental challenges due to persistent waste. Traditional mechanical recycling methods have proven insufficient in addressing these issues. This review explores recent advancements in polymer recycling, focusing on chemical and biological processes, such as pyrolysis, depolymerization, and enzyme-based degradation, which offer more efficient and sustainable alternatives. Within the framework of a circular economy, the review also examines strategies like closed-loop and circular plastic economies. These developments represent critical steps toward creating more sustainable plastic recycling practices. The final chapter includes the Quarterly Report 2024 on recycling plastics, providing an up-to-date overview of the current state of plastic recycling and its recent trends.

## 1. Introduction

The absence of plastics or synthetic organic polymers from modern life would be difficult to envision despite their large-scale manufacturing and integration being relatively recent advancements [1]. Early synthetic polymers, such as Bakelite, emerged in the early 20th century, but it was not until later that those plastics gained widespread application beyond specific sectors. Since then, the expansion of plastic production has been unprecedented, outpacing the growth of many other engineered materials, like steel and cement, extensively employed in construction, and continues to exhibit substantial industrial dominance, representing notable deviations from this trend [2,3].

However, in recent years, advancements in material science have facilitated the creation of innovative materials, which are increasingly replacing conventional metals and alloys in various engineering applications [4]. By combining materials with distinct characteristics, composite materials have emerged. Among these, polymer-based composites have gained considerable traction in industries due to their favorable strength-to-weight ratio [5,6]. These materials are widely employed in sectors such as aerospace and automotive engineering, where their mechanical properties, such as corrosion resistance and fatigue strength, are of paramount importance [7,8].

Polymer composites, often reinforced with carbon fibers (CFs) and glass fibers (GFs), have become integral to numerous engineering applications. In the aerospace sector, for instance, composite materials now constitute over 50% of the structure in aircraft. Carbon fiber-reinforced polymer (CFRP) and glass fiber-reinforced polymer (GFRP) are particularly prevalent, as exemplified by Boeing 787′s use of approximately 32 tons of composite materials [9,10]. Thermoset matrices, primarily epoxy resins, in these composites further enhance their durability, providing stability against environmental factors like humidity and temperature fluctuations. However, the increasing use of these materials raises significant concerns regarding plastic waste at the end of their lifecycle, driving ongoing research into waste management solutions [11,12].

Currently, the contribution of recycling to overall value generation remains relatively low, leading to the limited reintegration of significant quantities of used plastics and synthetic textiles into the economic cycle. Additionally, unlike metals and ceramics, the recycling of polymers typically results in a reduction in material properties. However, this does not preclude the potential for advancements that could enhance the quality of recycled polymer products to meet desired standards [13,14]. In the manufacturing process, a distinct category of solid plastic waste (SPW) emerges, classified as post-industrial (PI) waste, which does not reach the consumer stage. PI waste typically includes materials such as injection molding runners, production transition scraps, defective items, and various trimmings [15]. This waste type is advantageous due to its general lack of contamination and the known polymer composition. PI waste streams often consist of a single polymer type or mono-material, meaning they are not mixed with other polymers or non-polymeric substances [16].

Consequently, PI waste is typically considered a higher-grade polymer waste, making it more suitable for recycling. Conversely, at the end of their lifecycle, products become post-consumer (PC) waste. The collection and sorting processes for PC plastic waste vary by region, with some areas enforcing more stringent protocols than others. In many instances, PC waste includes mixed plastics with indeterminate compositions and may be contaminated by organic substances (e.g., food residues) or inorganic materials (e.g., paper), making recycling more complex compared to PI waste [17,18].

In addition, strict regulations imposed by the European Commission regarding the management of construction debris, end-of-life vehicles, and electronic waste are driving industries that utilize composites to investigate innovative and more efficient recycling methods for fiber-reinforced polymer (FRP) waste [19,20]. Recycling involves the repurposing of discarded materials, often requiring various processes to recover waste or convert it into new products, raw materials, or components. A circular economy, sometimes referred to as zero-waste manufacturing, represents an industrial paradigm where products are remanufactured, reused, and recycled after reaching their end-of-life (EOL) stage [21]. In modern industry, a key objective is to enhance circular economy designs through closed-loop recycling systems [22,23]. This approach is particularly advantageous for composite materials, as their physical properties are well-suited for efficient reprocessing. Ultimately, the circular economy not only mitigates the generation of hazardous materials and waste but also facilitates the production of goods with desired mechanical properties [24].

Scientists are advancing innovative technologies aimed at enhancing the efficiency of recycling and upcycling processes. A major focus of current research is the development of innovative recycling technologies, including thermomechanical processing, chemical recycling (e.g., glycolysis, pyrolysis), and biological depolymerization using enzymes and microorganisms [25]. While these technologies hold promise, challenges persist in material separation, economic feasibility, and societal acceptance [26].

The novelty of this review lies in its comprehensive analysis of state-of-the-art recycling technologies, with an emphasis on the latest innovations in polymer science and their industrial applications. Unlike previous studies, this review provides an updated assessment of emerging trends, regional case studies, and the scalability of advanced recycling methods. By addressing both technical and economic barriers, this study highlights opportunities for sustainable waste management and the integration of circular economy principles in polymer recycling. This review is supplemented by the Quarterly Report—Q1/2024, so the insights presented herein offer a valuable resource for researchers, policymakers, and industry professionals striving to mitigate plastic waste accumulation while maximizing material recovery efficiency.

## 2. Advancing Towards a Circular Plastics Economy

**Figure 1:**
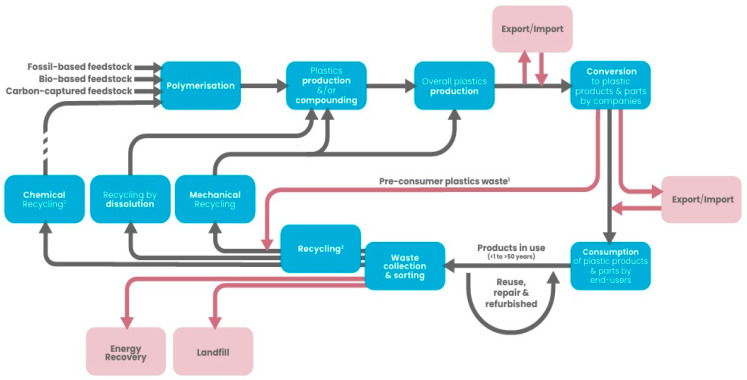
Circular plastic economy [27].

The circular economy for plastics (Figure 1) is a sustainable framework designed to extend the lifecycle of plastic materials [15]. This model emphasizes the reduction in plastic usage, alongside strategies for reusing and recycling plastics at the end of their functional life. It helps preserve the value of plastic waste as a resource, while simultaneously minimizing CO_2_ emissions and preventing plastics from being disposed of in landfills, incinerated, or contributing to marine pollution [27,28,29].

The 2022 report highlights that adopting circularity is the most rapid, cost-effective, and dependable strategy for diminishing plastic waste and lowering greenhouse gas (GHG) emissions within the plastics sector. Developing a circular economy for plastics is integral to the European Union’s Plastics Strategy and is essential to both the Circular Economy Action Plan and the Green Deal. A critical aspect of achieving a circular plastics economy is the need to significantly decrease Europe’s reliance on fossil-derived feedstocks and to transition towards circular alternatives. Such alternatives encompass recycled plastic waste, sustainably sourced bio-based materials, and CO_2_ from industrial activities [27,28].

The work plan on plastics was highlighted as a key priority within the “Closing the Loop” Action Plan for the Circular Economy [30]. While the Circular Economy Package established broad targets related to recycling rates and landfill reduction, the European Plastics Strategy specifically focuses on the packaging sector, providing a more targeted approach to addressing plastic waste and sustainability in this industry [31,32].

**Figure 2:**
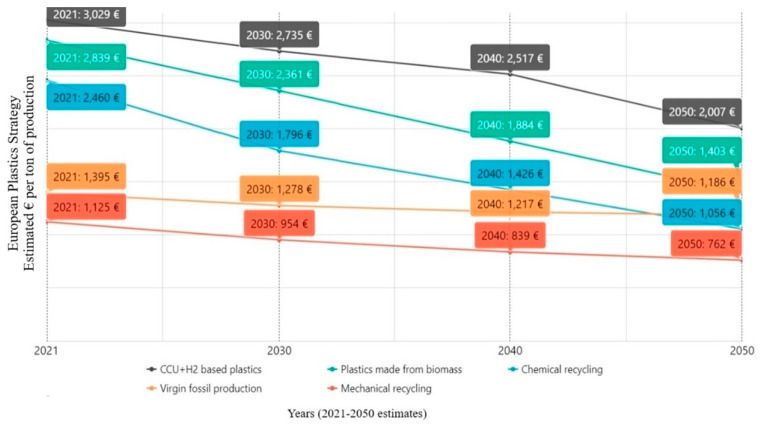
Estimated in euros per ton of production for the period 2021–2050 [33].

In 2023, European plastics manufacturers introduced the Plastics Transition roadmap [33] to guide the industry’s shift toward sustainability. The roadmap outlines a strategy to cut greenhouse gas emissions from the plastics sector by 28% by 2030, with the ultimate goal of achieving net-zero emissions by 2050 [32]. It also forecasts the progressive replacement of fossil-based plastics, estimating that circular plastics could fulfill 25% of European demand by 2030 and 65% by 2050 (Figure 2).

Achieving these targets is expected to require at least EUR 235 billion in additional investment and operational costs. The roadmap identifies key drivers and supportive measures and outlines specific short- and medium-term actions to accelerate the transition to a circular plastics economy for industry, policymakers, and the entire value chain [27].

## 3. Plastic Polymers: Types and Industrial Applications

In the context of polymer recycling, understanding the diversity of plastic polymers and their industrial applications is essential [34,35]. This chapter systematically categorizes the most prevalent and widely used plastic polymers, focusing on their key properties, production volumes, and the sectors where they are predominantly applied. By examining the specific applications of these polymers, we can better appreciate the challenges and opportunities in recycling each type, paving the way for improved recycling technologies and practices.

Given the wide range of polymer-based materials present both in commercial use and as waste, plastics are generally classified into two primary categories: thermosetting and thermoplastic [36]. Thermosetting polymers, characterized by their long molecular chains, undergo irreversible curing and cannot be reprocessed after initial use. In contrast, thermoplastics consist of shorter molecular links and can be re-melted and reshaped, granting them a degree of recyclability [37]. Plastics are generally categorized into six principal groups: Low-Density Polyethylene (LDPE), High-Density Polyethylene (HDPE), Polypropylene (PP), Polystyrene (PS), Polyethylene Terephthalate (PET), and Polyvinyl Chloride (PVC). These classifications represent the most common types of polymer materials used across various industries [38,39].

**LDPE—Low-Density Polyethylene (Thermoplastic)**

**Figure 3:**
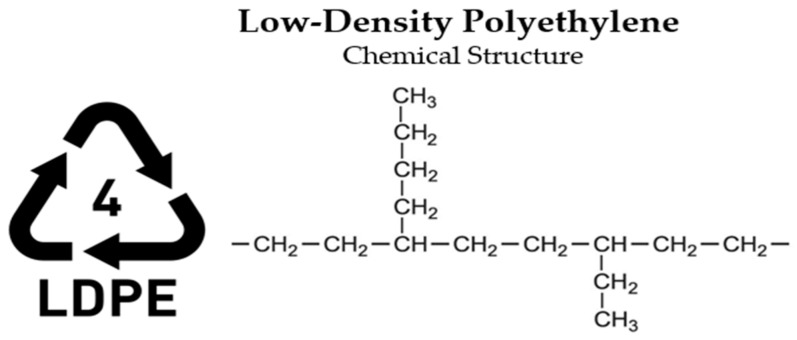
Labeling and chemical Structure of low-density polyethylene (LDPE).

Applications: LDPE (Figure 3) is characterized by its branched molecular structure, which results in lower density and weaker intermolecular forces compared to high-density polyethylene (HDPE). These properties make it an ideal material for various applications, such as packaging for computer components, including hard disk drives, graphics cards, and optical disc drives, as well as trays. Additionally, low-density polyethylene (LDPE) is available in two forms: recycled granules (R), derived from leftover bottle caps and certain types of containers, and virgin granules (V), used for their production. Due to its flexibility, chemical resistance, and good electrical insulation properties, LDPE is also widely employed in the production of consumer goods and industrial products [40].

**Thermosetting: Vinyl ester**

Applications: The marine sector, FRP (fiberglass reinforced plastic) tanks and vessels, lamination processes, and kit airplanes like Glasair and Glastar [38]. Vinyl ester is known for its excellent corrosion resistance, high mechanical strength, and good adhesion properties. These characteristics make it a reliable choice for structural and load-bearing applications in demanding environments.

**HDPE—High-Density Polyethylene (Thermoplastic)**

**Figure 4:**
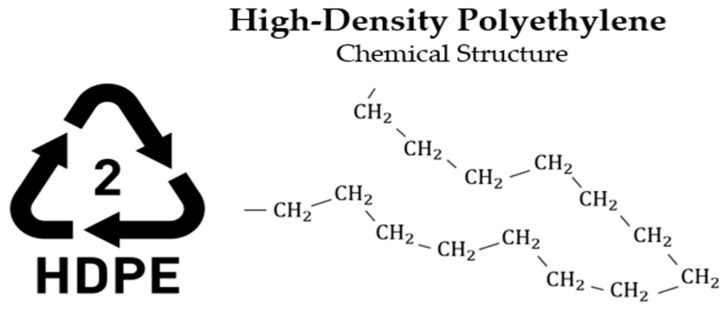
Labeling and chemical structure of high-density polyethylene (HDPE).

Applications: High-density polyethylene (HDPE) (Figure 4) is classified into two forms: recycled granules (R), obtained from post-consumer waste such as containers for cleaning agents, shampoos, and milk bottles, and virgin granules (V), which are employed in the synthesis of new HDPE products. Items such as toys, kitchenware, films, bottles, piping, processing machinery, as well as wire, cable insulation materials, and the packaging sector, which represents the most significant application of plastics, comprising 31% of total usage and achieving a market size of over 236 billion euros in 2022 [41].

**Thermosetting: Phenol formaldehyde resin**

Applications: Billiard balls, laboratory work surfaces, coatings and adhesives, electronic circuit boards, and fiberglass fabrics, among other applications. It is valued for its high thermal stability, chemical resistance, and durability.

**PP—Polypropylene (Thermoplastic)**

**Figure 5:**
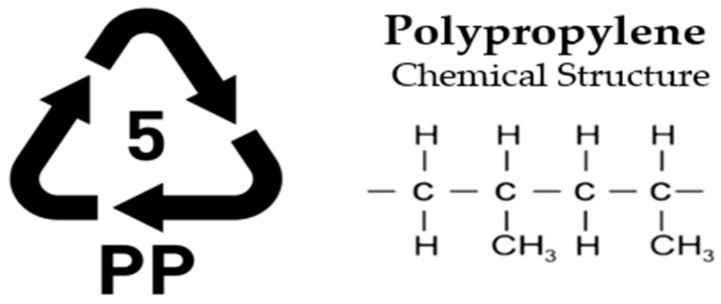
Labeling and chemical structure of polypropylene (PP).

Polypropylene (Figure 5) is known for its high chemical resistance, low moisture absorption, and good electrical insulating properties, making it suitable for a wide range of industrial and consumer applications. Its semi-crystalline structure provides a balance of rigidity and flexibility while maintaining excellent durability and impact resistance. Additionally, polypropylene’s recyclability aligns with sustainable material practices, contributing to waste reduction in manufacturing and construction sectors. These properties enable its use in various applications, such as biaxially oriented polypropylene (BOPP), transparent packaging bags, carpets, rugs, mats, and recycled aggregate concrete, where the static elastic modulus reduces as the fiber volume fraction increases [42].

**Thermosetting: Silicon**

Applications: Sealants, adhesives, lubricants, pharmaceuticals, cooking implements, thermal and electrical insulation materials, and silicone grease. Silicon is valued for its exceptional temperature resistance, weather stability, and electrical insulating properties. These attributes make it a versatile material in both industrial and consumer applications requiring durability and reliability.

**PS—Polystyrene (Thermoplastic)**

**Figure 6:**
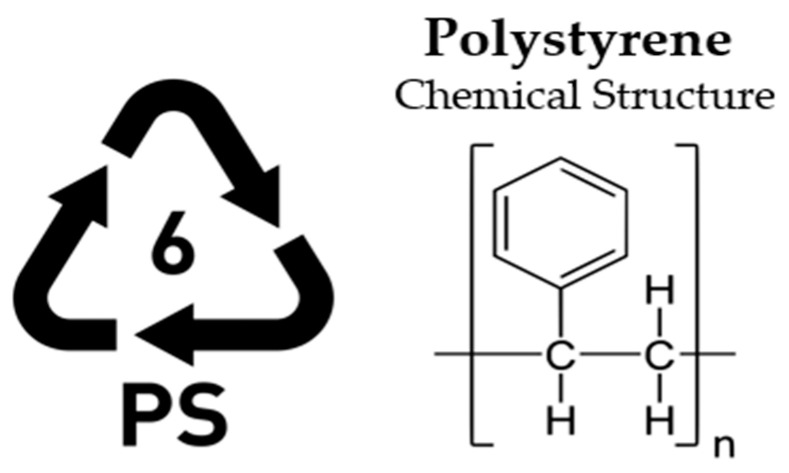
Labeling and chemical Structure of polystyrene (PS).

Applications: Single-use plastic cutlery and dinnerware, CD cases, housings for smoke detectors, license plates, and frames. Significantly, the most extensive expanded polystyrene (PS) (Figure 6) recycling facilities are predominantly located in north-western Europe. A new methodology was also created for developing triboelectric generators utilizing a single triboelectric polymer (PS) [43].

**Thermosetting: Polyester**

Applications: Staple fibers (PSF), containers for carbonated soft drinks (CSD), water, beer, juice, and detergents, as well as industrial yarns and tire reinforcement cords.

**PET—Polyethylene Terephthalate (Thermoplastic)**

**Figure 7:**
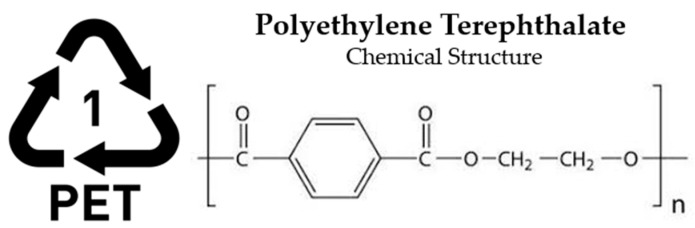
Labeling and chemical structure of polyethylene terephthalate (PET).

Applications: Polyethylene terephthalate (PET) (Figure 7), designated with a resin identification code #1, ranks as the third most prevalent polymer in the packaging industry. It is the primary material used for beverage containers, contributing to nearly 16% of total plastic consumption in the European packaging sector. Also, it has applications such as packaging films, PET bottles, carpet yarns, engineering plastics, filaments, non-woven materials, packaging straps, and staple fibers [44,45].

**Thermosetting: Urea—Formaldehyde**

Applications: Wall cavity fillers, agricultural products, decorative laminates, textiles, paper materials, foundry sand molds, wrinkle-resistant fabrics, cotton blends, rayon, corduroy, and others.

**PVC—Polyvinyl chloride (Thermoplastic)**

**Figure 8:**
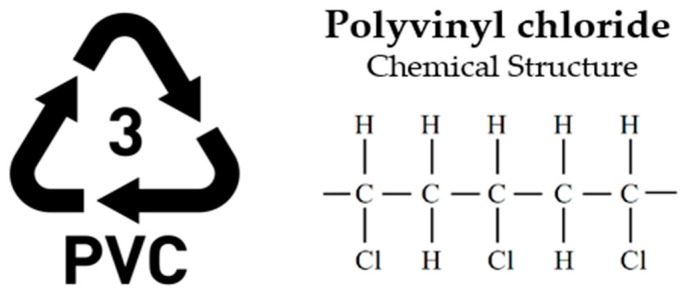
Labeling and chemical structure of polyvinyl chloride (PVC).

Applications: The physical properties of PVC (Figure 8), such as flexibility, rigidity, and color, are influenced by the specific additives employed, including lubricants, plasticizers, and pigments, which are tailored to achieve the desired performance in the final product. This versatility enables PVC to be extensively utilized across various industries, such as construction, electrical systems, and consumer goods.

**Figure 9:**
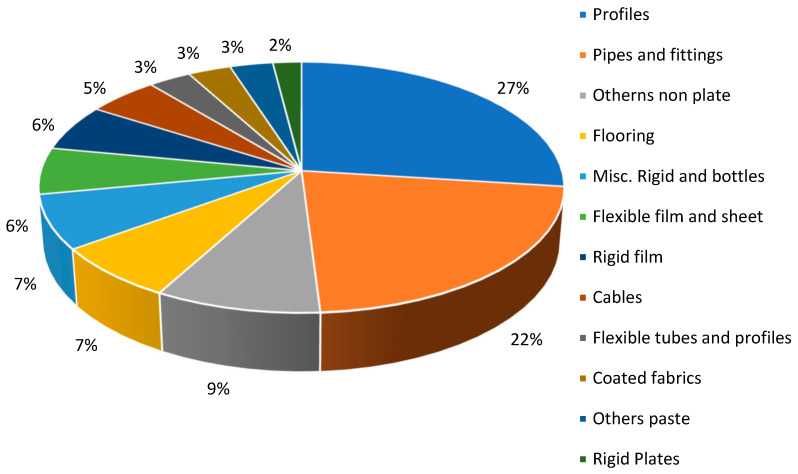
The diverse applications of the 6.5 million tons of PVC produced annually across the EU-27, Norway, the UK, and Switzerland [47].

Notably, polyvinyl chloride (PVC) production in the EU-27, Norway, the UK, and Switzerland accounts for 6.5 million tons (Figure 9), reflecting its significant role in the materials industry. PVC is widely used in construction, automotive, and electrical applications due to its durability, chemical resistance, and cost-effectiveness. The high production volume also highlights the ongoing demand and economic importance of PVC in various industrial sectors, as well as efforts to improve its sustainability through recycling and eco-friendly production processes [46].

**Thermosetting: Bakelite**

Applications: Electrical systems include non-conductive components found in telephones, radios, and various electronic devices, such as bases and sockets for light bulbs and vacuum tubes, supports for different electrical elements, distributor caps in automobiles, and insulators.

Temperature influences polymer behavior, leading to effects such as melting, degradation, and morphological changes (Table 1 and Table 2) [48]. It also impacts mechanical properties by increasing polymer chain mobility and free volume. These effects are characterized by two key temperatures: glass transition temperature, related to the amorphous regions, and melting temperature, associated with crystalline regions. Below the glass transition temperature, polymer chains remain in a rigid, glassy state, with limited movement. When heated above this threshold, polymers enter a rubbery state, where chain mobility increases, making the material soft and flexible. The heating process affects semi-crystalline polymers more complexly, where the amorphous regions soften first, providing initial flexibility, while crystalline regions retain their structure until the melting point. Furthermore, exceeding thermal stability thresholds can induce oxidative reactions, accelerating degradation. Prolonged heating of amorphous plastics causes them to soften and transition into a viscous liquid. For crystalline polymers, the melting temperature represents the point at which crystalline regions break down, leading to a dramatic loss in rigidity and strength. In semi-crystalline polymers, these transitions coexist, with the amorphous regions softening first, followed by the melting of crystalline structures. Additionally, heating polymers beyond a critical temperature can result in thermal degradation, where chemical bonds break, leading to molecular weight reduction and potential loss of mechanical properties. This degradation can manifest as discoloration, brittleness, or loss of elasticity, depending on the polymer type and exposure duration [49].

These tables present the physical and mechanical characteristics of primary plastic materials, highlighting key properties that influence their performance and applications. Table 1 outlines material-specific attributes, such as morphology, density etc., while Table 2 provides insights into initial degradation temperature, tensile strength, etc.

## 4. Current Advances in Plastic Recycling: Methods and Trends

**Figure 10:**
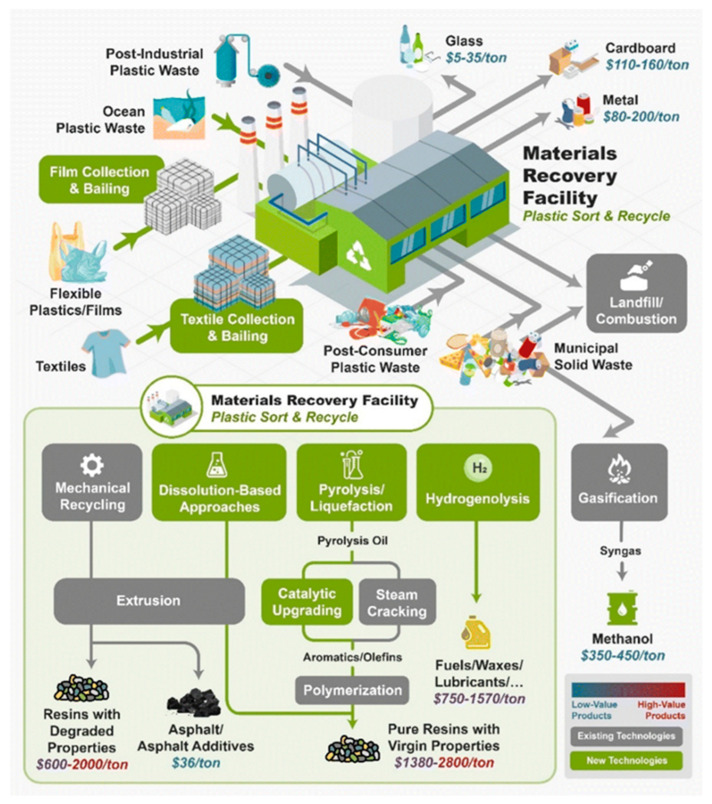
Overview of the current plastic waste management system [49].

This chapter provides a comprehensive overview of the latest advancements in polymer recycling, highlighting both traditional and emerging methods (Figure 10).

Mechanical recycling remains one of the most widely utilized methods for plastic waste management due to its cost-effectiveness and accessibility. However, it is often hindered by significant limitations, including material degradation, reduced tensile strength, loss of molecular integrity, and decreased performance characteristics, which compromise the usability of the recycled product. To overcome these constraints, chemical recycling techniques have emerged as a crucial area of research and industrial development. Methods such as depolymerization, pyrolysis, gas-phase cracking, and solvent-based purification enable the breakdown of plastic materials into their original monomeric components, facilitating the production of high-quality resins that meet stringent performance criteria and maintain mechanical properties comparable to virgin materials [49].

Recent studies are also exploring advanced catalytic recycling processes, utilizing heterogeneous and homogeneous catalysts to optimize reaction efficiency, minimize energy consumption, and improve material yield. Furthermore, enzyme-driven recycling technologies offer an eco-friendly approach by leveraging biological processes to selectively degrade complex polymers under mild conditions, reducing reliance on high-temperature or high-pressure methods. Supercritical fluid technologies are also being investigated for their potential in separating mixed polymer streams with high purity and minimal degradation.

### 4.1. Primary Mechanical Recycling: Fundamentals and Techniques

**Figure 11:**
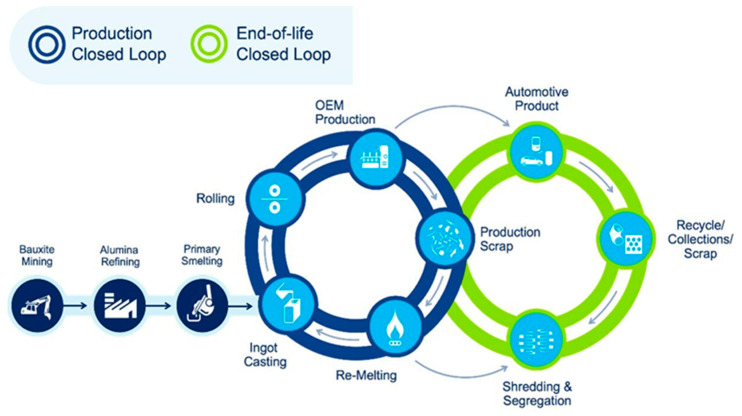
Advantages of closed-loop and end-of-life recycling systems for automotive manufacturers [52].

Primary mechanical recycling, also known as closed-loop recycling (Figure 11), involves the reprocessing of uncontaminated polymer waste directly into new products, with minimal degradation in material properties. This method is primarily employed by manufacturers to recycle post-industrial waste, making it a cost-efficient and resource-conserving technique [50]. Its use for post-consumer plastic waste is possible but presents significant challenges, such as the necessity for precise waste segregation and extensive manual sorting. These additional processing steps contribute to increased operational costs, thereby reducing its attractiveness for post-consumer applications [51].

The recycling process typically begins with the mechanical breakdown of polymeric materials, such as shredding, crushing, or milling. This size reduction enhances the uniformity of the material and facilitates its reintroduction into production cycles, often in combination with fresh polymers or additives. The homogeneity achieved through these methods allows for better integration of recycled polymers into standardized manufacturing processes. Furthermore, mechanical purification, sometimes supplemented with cleaning procedures, is critical to ensure the quality of the recycled material and to prevent defects in the resulting products [53].

Recycled polymers, also known as recyclates, can be reshaped into new forms after undergoing a melting process. The most widely used techniques for reprocessing mechanical recyclates include injection molding, extrusion, rotational molding, and heat pressing. These methods are primarily applicable to thermoplastic polymers, such as polypropylene (PP), polyethylene (PE), polyethylene terephthalate (PET), and polyvinyl chloride (PVC) [54], as these materials can be repeatedly melted and reshaped without significant loss of properties. However, repeated recycling cycles may lead to gradual degradation of polymer chains, affecting mechanical and thermal performance.

Efficient closed-loop recycling systems operate based on several critical factors:

- Recovered materials are reintroduced rapidly into the manufacturing process.
- Contaminants can either be effectively removed or are inconsequential to the final product, particularly when incorporated into specific layers of the recycled material.
- The polymer retains sufficient thermal stability to withstand subsequent high-temperature processing stages.
- The reprocessed materials can be handled similarly to virgin polymers, allowing seamless integration into conventional production workflows [55,56].

### 4.2. Secondary Mechanical Recycling: Techniques and Material Integrity

**Figure 12:**
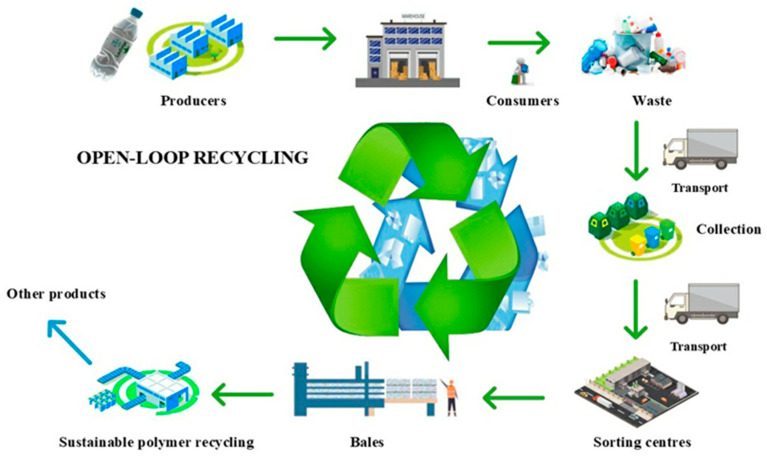
The principle of open-loop recycling/secondary recycling.

Secondary recycling, often referred to as downcycling or open-loop recycling (Figure 12), involves the recovery, sorting, and processing of waste thermoplastics into materials of lower quality or reduced value compared to the original polymers. Unlike primary recycling, where the material properties are largely retained, secondary recycling is characterized by a degradation in the polymer’s mechanical and physical properties, limiting its suitability for high-performance applications. Common examples of products derived from secondary recycling include composite materials like plastic wood, packaging, and various construction-related products [57].

The exact composition and purity of end-of-life (EOL) and post-consumer (PC) plastic waste streams are frequently unknown, making secondary recycling necessary. This process requires extensive separation and purification efforts that are not as critical in primary recycling. Although secondary recycling predominantly handles thermoplastics, the processing can lead to chain scissions in the polymer matrix, primarily due to exposure to moisture and trace acidic substances. This results in a decrease in molecular weight and a subsequent reduction in mechanical performance. However, certain strategies, such as thorough drying, vacuum degassing, and the use of stabilizing additives, can mitigate some of these adverse effects [58].

Another significant factor affecting the mechanical properties after recycling is the contamination of the base polymer with other incompatible polymers. Since most polymers are immiscible, the resulting blends often exhibit inferior mechanical properties compared to their pure counterparts [59]. For instance, the inclusion of PET in PVC leads to the formation of solid PET inclusions within the PVC matrix, greatly diminishing the material’s overall performance and limiting its application [60].

To address these challenges, efficient separation methods are critical before reintegration into new products. Advanced techniques, such as Fourier-transform infrared (FTIR) spectroscopy and near-infrared (NIR) spectroscopy, are frequently employed to identify polymer types, while optical color recognition systems are used to distinguish clear from colored plastics [60]. Additionally, X-ray detection is effective in isolating PVC to prevent the release of HCl during high-temperature processing. Newer methods, such as laser sorting, have proven valuable for separating different types of plastic in waste streams from electronic equipment and automotive parts, with electrostatic detection also emerging as a promising technology [61].

As in primary recycling, the waste materials are usually ground before any purification step, which can occur either before or after grinding. These materials are then melted and reintegrated into the production process. However, the efficiency of secondary recycling depends on several key factors:

- Availability and logistics related to waste materials, including collection, storage, and transport costs.
- The physical form and size of the waste, whether it be flakes, fibers, or other shapes.
- The complexity of the material composition, particularly if the waste contains multiple components with varying melting points.
- The price differential between virgin and recycled polymers can make secondary recycling of high-value technical polymers financially attractive.
- The presence of additives that influence the recyclate’s characteristics, such as odor or color, may restrict its use in end products unless purification, deodorizing, or decolorizing processes are applied.
- The availability and costs of advanced separation, detection, and purification technologies.
- Environmental considerations include dust generation, noise from mechanical processing, energy consumption, and the potential toxicity of solvents used in purification processes.

By carefully managing these factors, secondary recycling can provide a viable option for reducing plastic waste, though it is generally best suited for lower-grade applications. This limitation arises due to the gradual degradation of polymer chains during reprocessing, which affects material properties [62].

#### Utilization of Recycled Polymers as Fillers in Composite Materials

The integration of functionalized thermoplastic waste materials as reinforcements in thermoset polymers presents a significant opportunity to enhance composite performance while addressing sustainability challenges associated with plastic waste management. Polymer matrix composites are already widely employed across various industries due to their outstanding material properties, such as high specific strength, excellent impact resistance, superior abrasion resistance, and substantial chemical and corrosion resistance. These composites typically consist of a polymer matrix combined with either organic or inorganic reinforcements, which can be natural or synthetic, and serve to improve mechanical, thermal, and economic performance [63].

Functionalizing recycled thermoplastics enhances their compatibility and dispersion within the thermoset matrix, thereby strengthening interfacial adhesion and overall composite integrity. As industries seek high-performance composites for applications in sectors ranging from aerospace, automotive, and electronics to healthcare, civil engineering, and marine engineering, it becomes crucial to explore the performance characteristics of composites reinforced with waste materials [64]. This research aims to systematically review existing literature on the physical, mechanical, and wear properties of composites incorporating functionalized thermoplastic waste, emphasizing their role in advancing sustainable material development, environmental responsibility, and performance optimization under diverse operational conditions [65].

Recent research investigated the potential of utilizing recycled thermoplastic materials as fillers in thermoset composites [66], focusing on two specific types of polymer waste: poly(ethylene terephthalate) (PET) and polycarbonate-based Colombian Resin (CR-39). In this study, PET was employed to synthesize a thermoset unsaturated polyester resin (UPR), while CR-39 served as a reinforcing element within the UPR matrix. Before integration, CR-39 particles underwent surface modification through oxidation and chemical activation using amines to improve their compatibility with the matrix. The findings demonstrated that properly functionalized recycled fillers significantly enhanced the mechanical performance of the composite, particularly tensile and flexural strength, with optimal improvements observed at specific concentrations of activated particles. Activation energy calculations, based on the Arrhenius equation, indicated that the presence of these fillers increases the energy barrier required for macromolecular viscoelastic relaxation.

This study underscores the feasibility of using recycled thermoplastic materials as effective reinforcements in composite matrices, highlighting their ability to improve mechanical and thermal properties while simultaneously contributing to sustainable waste management and eco-friendly material development [66]. Moreover, incorporating these materials reduces environmental pollution by mitigating plastic waste accumulation and minimizing ecological impact. From an economic perspective, using recycled fillers significantly lowers material costs, enhancing the cost efficiency of composite production. However, challenges remain, such as achieving uniform dispersion of recycled fillers and maintaining long-term material stability.

### 4.3. Chemical Recycling Solutions for End-of-Life Polymers

“Chemical recycling is a game-changer and a key building block of the circular economy—not only in Europe. This increased investment confirms the determination of the industry to address the problem of plastic waste and supports the EU Green Deal’s climate and sustainability ambitions. However, this is just a starting point, and sizeable investments are still needed to fully capture the value of this technology ” [67].

**Figure 13:**
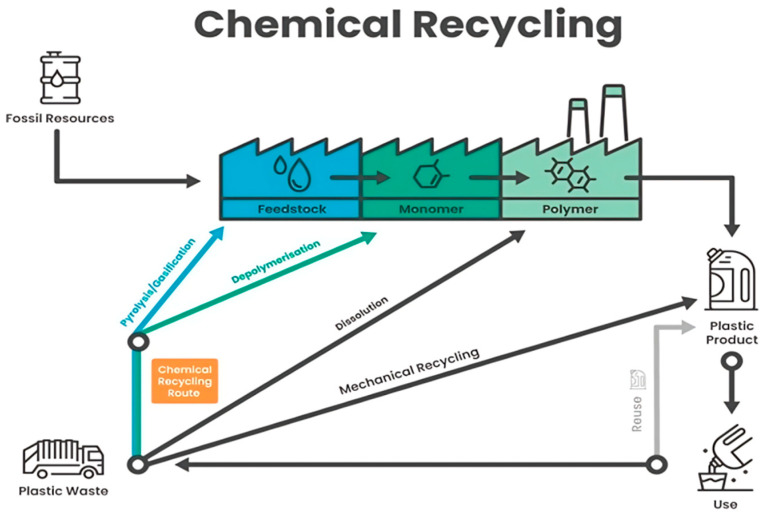
Flow of chemical recycling process of polymers [73].

Chemical recycling (Figure 13) encompasses a range of innovative technologies within the waste management sector that facilitate the recycling of plastics that are either challenging to process or not economically viable through mechanical methods [68,69,70]. Serving as a complementary approach to mechanical recycling, chemical recycling enables the recovery of value from polymers that traditional methods can no longer process efficiently [71]. This process not only provides an alternative to landfilling and incineration, especially for difficult-to-recycle plastics such as films and multi-layered laminates but also yields high-purity raw materials for the industry. As a result, it enhances the potential for producing food-grade products from post-consumer waste. A recycling method refers to any technology that employs processes or chemical agents to alter the molecular structure of polymers [72]. These technologies can be classified into three main categories, depending on the stage at which their outputs re-enter the plastics supply chain.

#### 4.3.1. Purification

Solvent-based purification is a crucial method for obtaining high-purity plastic materials, as it leverages the selective solubility of polymers in specific solvents or solvent mixtures. The process typically begins by dissolving the target plastic in a chosen solvent, which allows for the separation of additives, stabilizers, and contaminants. During selective crystallization, the solvent’s affinity ensures that only the desired polymer remains, effectively isolating it from unwanted components. A solvent with a high selectivity is essential to achieve this separation efficiently and minimize contamination. Additionally, temperature control and solvent choice play a significant role in maintaining the polymer’s mechanical and chemical integrity. The purified material, free of additives and contaminants, can then be processed back into high-value applications, such as consumer goods, industrial components, and construction materials, ensuring both material sustainability and economic viability. Target feedstocks for this purification method include polyvinyl chloride (PVC), polystyrene (PS), polyethylene (PE), and polypropylene (PP) [73].

#### 4.3.2. Depolymerization

Depolymerization, often termed chemolysis, involves the breakdown of polymer chains into individual monomers or smaller units known as oligomers, effectively reversing the polymerization process. The monomers produced are structurally identical to those used in the original polymer formation, which enables the production of recycled plastics with qualities comparable to those made from virgin materials. A key limitation of this process is that it is primarily applicable to condensation polymers, such as polyamides and PET. It is not effective for most additional polymers, including PP, PE, and PVC, which constitute the bulk of plastic waste streams. Target Feedstock: Polycondensates, which include polyesters (PET), polyamides (PA) and Polyurethanes. Several industrial facilities are actively engaged in PET degradation, predominantly utilizing methanolysis and glycolysis processes [74].

In contrast, hydrolytic methods are less mature, primarily limited to laboratory and pilot-scale operations, though multiple projects are underway to bring these technologies to commercial scale shortly. Processes based on ammonolysis and aminolysis are less widely adopted and remain underdeveloped. Among chemolysis methods, glycolysis and hydrolysis are the most employed for reversing the polymerization of polyurethane. The depolymerization of polyamides, on the other hand, is primarily achieved through hydrolysis. Improving catalyst efficiency and optimizing reaction conditions are crucial for scaling chemolytic processes economically. Advances in solvent recovery and energy integration also play a significant role in enhancing sustainability [69].

##### Depolymerization of PET in SPI Code 1

Polyethylene terephthalate (PET) is a commonly employed thermoplastic known for its strong resistance to chemicals and impact at ambient temperatures, predominantly used in consumer packaging, such as beverage bottles. While PET itself is not a significant environmental hazard, its accumulation in landfills, particularly from discarded water bottles, contributes substantially to environmental pollution, whereas recycling PET conserves petrochemical resources and energy [75,76].

PET recycling primarily occurs through two traditional methods: mechanical and chemical approaches. Mechanical recycling, while cost-effective, often results in materials with diminished quality, making it unsuitable for food and beverage packaging due to contamination and polymer degradation [77]. In contrast, chemical recycling ensures higher quality repolymerized products but remains economically less viable due to the high costs of raw materials and starting monomers. Industrial chemical recycling methods typically involve breaking ester groups with agents like glycols, methanol, or water, often requiring elevated temperatures and catalysts such as cobalt acetate or lithium hydroxide [78].

##### Depolymerization of HDPE, LPDE and PP for SPI Code 2, 4, and 5

Polyolefins, such as HDPE, LDPE, and PP, represent the predominant category of synthetic plastics and are crucial commodities in polymer markets. The strong C-C and C-H bonds in polyethylene and polypropylene contribute to their chemical stability, making low-energy depolymerization difficult [79]. Plastic fuels derived from polyolefins benefit from high carbon and hydrogen content, resulting in fuels that are non-corrosive, non-acidic, and possess a higher heating value due to the absence of water. Pyrolysis and supercritical water depolymerization are the primary methods for converting polyolefins into fuels [80]. In supercritical water depolymerization, high temperature and pressure alter water properties (density, viscosity, solvation power), facilitating rapid and selective depolymerization through H abstraction and β-scission mechanisms.

H-abstraction: *R*_1_ + *M k_β_ R*_1_*H* + *R*_1_ (1)

*β*-*scission*: *R*_1_ *k_β_* *R*_1_ + *O_l_* (2)

Supercritical water depolymerization operates under high temperature (≥374.15 °C) and pressure (≥22.129 MPa) conditions, where changes in properties like density, viscosity, and solvation power enhance reaction efficiency. This process enables rapid and selective conversion of organic waste into oil, outperforming traditional methods. Studies, such as Watanabe et al., have shown successful conversion of LDPE under specific conditions (400 °C, >30 MPa, 30 min), where mechanisms like H abstraction and β-scission drive the depolymerization reactions, resulting in the breakdown of polyethylene into smaller molecular fragments [81].

##### Depolymerization of PVC for SPI Code 3

Polyvinyl chloride (PVC) is a versatile plastic known for its stability under chemical and thermal conditions, capable of transitioning from flexible to rigid forms depending on the additives and plasticizers mixed with it. Despite its wide usage, PVC poses environmental challenges due to the release of harmful compounds like phthalates and chlorine-containing organics during degradation. Recycling PVC is hindered by HCl emissions, which corrode processing equipment, necessitating pretreatment steps (300 °C for 60 min) to reduce chlorine levels. Alternative approaches include using HCl scavengers, such as CaO in a ball mill, which produces a removable calcium salt by-product [82].

##### Depolymerization of PS for SPI Code 6

Polystyrene is a widely utilized material in packaging, available in both solid and foamed forms. The general-purpose variant is transparent, rigid, and brittle at ambient temperatures but becomes malleable above 100 °C. This thermal property enables applications such as Styrofoam, a lightweight, waterproof material, and expanded polystyrene (EPS), which is employed in insulation, flotation devices, and food containers. However, polystyrene’s environmental impact is exacerbated by its stability and low density, making landfill disposal problematic. Additionally, waste polystyrene often becomes contaminated with other plastics, complicating its recycling [83].

Traditional methods for polymer waste management, such as landfilling and incineration, have inherent limitations. The predominant recycling method for polystyrene involves thermal or thermocatalytic breakdown, producing a liquid oil composed mainly of C6–C12 aromatic hydrocarbons, gas, and solid remnants. A significant drawback of these chemical approaches is the formation of a heterogeneous liquid product containing various hydrocarbons, which has poor thermal-oxidative stability due to high concentrations of styrene and α-methylstyrene. When used as automotive fuel, the elevated aromatic content can cause engine carbon deposits. Though catalysts reduce olefinic compounds during decomposition, they also result in increased gas production and substantial coke formation, intensifying processing challenges [84].

#### 4.3.3. Feedstock Recycling—Pyrolysis

Feedstock recycling refers to thermal processes that break down polymers into simpler molecules, which can be used as raw materials. Pyrolysis and gasification are the two primary methods employed in this approach. The products generated from feedstock recycling, such as hydrocarbons or syngas, serve as fundamental chemicals requiring further processing to synthesize new polymers, so they provide adaptability into petrochemical manufacturing [85].

In the pyrolysis process, plastics undergo thermal degradation into a variety of basic hydrocarbons through heating in an oxygen-free environment, often referred to as thermal cracking. Subsequently, the hydrocarbon vapors can be separated via distillation, yielding products that range from heavy waxes and oils to lighter oils [86]. The distribution of products can be adjusted by varying the processing time and temperature, allowing for a shift from heavier to lighter outputs. Heavier by-products can also be reintegrated into the system for further cracking into lighter fractions. The products of pyrolysis can be processed similarly to crude oil, employing conventional refining methods to produce building blocks for polymer synthesis. Alternatively, they may be utilized directly as fuel [87].

**Figure 14:**
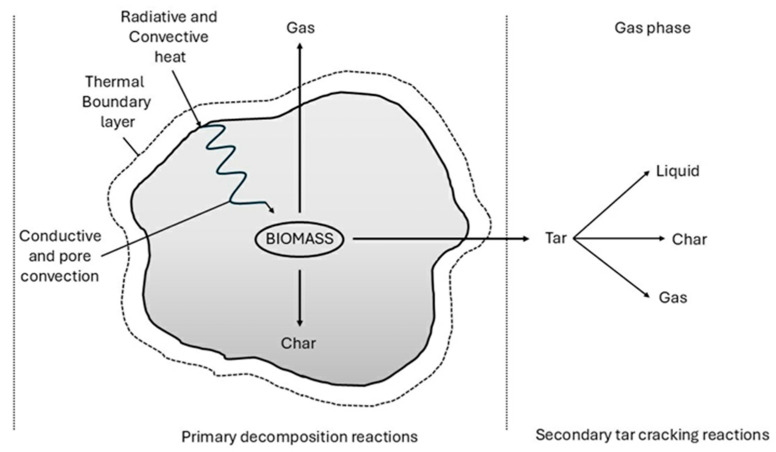
Schematic of the pyrolysis process within a biomass particle.

Upon rapid heating, the large molecular structures in biomass experience primary decomposition. The cracking process produces condensable vapors and solid char as by-products. These condensable vapors can undergo further secondary cracking, resulting in non-condensable gasses (CO, CO_2_, H_2_, and CH_4_), liquid products, and char (Figure 14). The decomposition mechanism involves both gas-phase and gas–solid thermal reactions. In the gas phase, the condensable vapors break down into smaller, non-condensable permanent gasses, such as CO and CO_2_, though these are of lesser significance in the pyrolysis process [87,88]. A general reaction scheme for pyrolysis can be expressed as follows:

(3) CnHmOp(biomass)→∑liquidHeatCxHyOz+∑gasCaHbOc+H2O+C(char)

Utilizing pyrolysis to generate feedstock to produce polyethylene and polypropylene addresses a significant processing gap, as these plastics cannot be depolymerized directly into their monomers. Additionally, the resulting materials would possess properties comparable to virgin polymers. The efficiency of pyrolysis can be improved through catalytic degradation, where a catalyst is employed to facilitate the cracking reactions. The introduction of a catalyst enables the reduction in reaction temperature and time, leading to a more concentrated product distribution in terms of carbon atom count and enhancing the yield of lighter hydrocarbons [89].

Historically, pyrolysis has been utilized commercially in the production of charcoal, as well as in the management of municipal solid waste and biomass. In the waste management sector, the pyrolysis of mixed plastics has been under development for the past two decades and is now transitioning into a commercial phase, with several operational plants and numerous additional industrial-scale facilities anticipated to be established in the coming years [90].

#### 4.3.4. Benefits of Chemical Recycling

Chemical recycling serves as a complementary method to other plastic recycling techniques, such as mechanical, dissolution, and organic recycling. It is particularly advantageous for handling complex plastic waste, including films and laminates, that would typically be destined for incineration or landfill. Given that 67.5% of post-consumer plastic waste in Europe is currently directed to landfill or energy recovery, chemical recycling presents significant opportunities for improvement. By breaking polymers down into their fundamental components, chemical recycling enables the creation of recycled plastics with properties comparable to virgin materials, making them suitable for high-performance applications, including food packaging [68].

**Figure 15:**
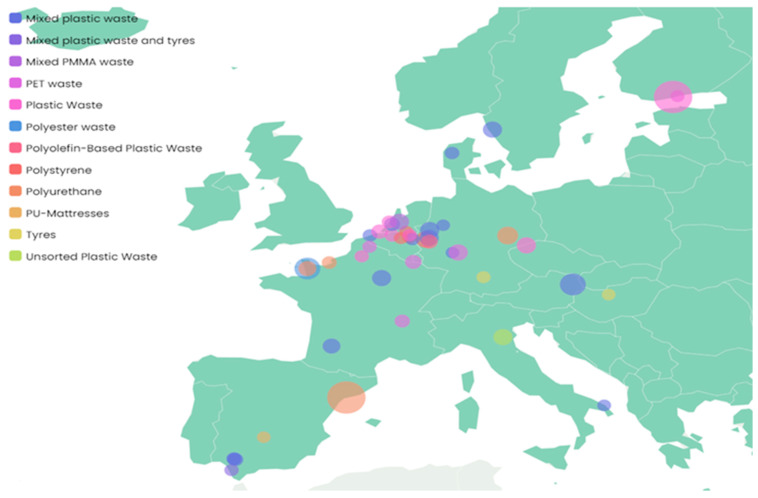
Planned investment in chemical recycling—data from announcements made by both member and non-member organizations of Plastics Europe [73].

The European Commission has outlined ambitious goals for plastic circularity (Figure 15). Revised waste directives set targets of reducing municipal waste landfilling to a maximum of 10% by 2035, achieving 50% recycling of plastic packaging by 2025, and 55% by 2030. In this context, chemical recycling plays a crucial role in minimizing waste and advancing the transition toward a circular plastics economy [68].

The economic evaluation of plastic chemical recycling still contains notable gaps, particularly in the areas of recovery and preparation. While substantial progress has been made in establishing the technical feasibility, environmental impact, and overall viability of various stages of chemical recycling, there remains limited economic assessment data due to the scarcity of commercial projects and comparative studies. However, this does not undermine the economic and societal potential of chemically recycled plastics. In North America (U.S. and Canada), integrating recycled plastics could represent a significant USD 120 billion market, yet only around 6% of this demand is currently met through technologies like depolymerization and solvolysis. Other thermochemical outputs, such as syngas, liquid fuels, and refined chemicals, are not included in these estimates [69].

Expanding the chemical recycling sector would improve the efficiency of plastic collection systems, which would, in turn, reduce recycling costs, potentially saving hundreds of euros per ton of plastic waste collected, as reported by the European Union. Additionally, this expansion would contribute to environmental sustainability by lowering CO_2_ emissions and decreasing reliance on fossil fuels. Utilizing recycled feedstocks with more stable market prices would strengthen the economic resilience of a circular economy [70].

Moreover, from a broader environmental perspective, chemical recycling addresses waste management challenges by transforming plastic waste into valuable products or energy sources, instead of relying on landfill disposal. This dual function—both treating waste and generating useful outputs—highlights the positive and negative impacts of waste activities on the environment [71]. A comprehensive life-cycle assessment (LCA) would provide a robust framework for comparing different recycling technologies (pyrolysis, solvolysis, gasification, etc.) and feedstock types (polyolefins, composites, PS, PVC, etc.) [72].

In conclusion, while the economic assessments of chemical recycling remain incomplete, the integration of recycled plastics into commercial markets offers substantial economic opportunities and significant environmental benefits. Strengthening collection infrastructure, supporting technological advancements, and prioritizing closed-loop and open-loop recycling pathways would enable a more sustainable, economically viable, and environmentally responsible system for plastic waste management.

### 4.4. Biodegradation of Polymers: Pathways to Sustainable Polymer Recycling

Comprehending the processes that govern plastic biodegradation is essential for establishing effective and feasible methodologies in the future [91].

**Figure 16:**
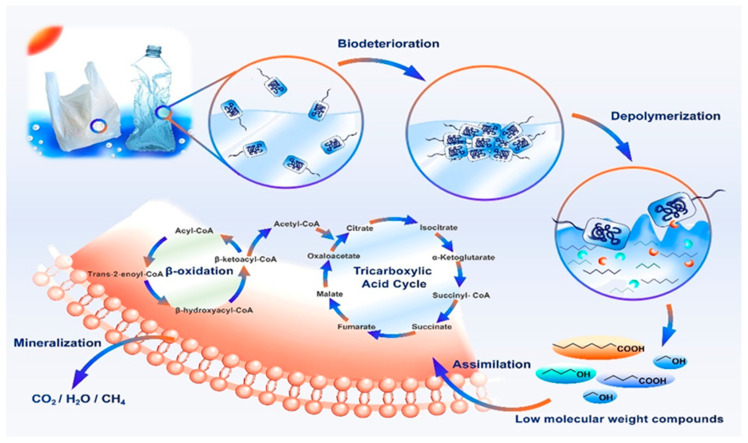
Depiction of possible mechanism involved in plastic biodegradation [93].

While the biodegradation of plastics remains incompletely understood and differs across various polymer types, current research categorizes the process into four main stages: biodeterioration, depolymerization, assimilation, and mineralization (Figure 16) [92].

Biodegradation, a process by which complex organic materials are broken down into simpler substances such as carbon dioxide, methane, water, minerals, and new biomass, occurs through the metabolic activity of microorganisms like bacteria and fungi [94]. These microorganisms initiate degradation by colonizing the surface of plastics, secreting a biofilm containing enzymes that fragment the long polymer chains into smaller segments. These shorter fragments are then transported into the microorganisms for further metabolism. However, the efficiency of this biodegradation process is determined by several factors. Among these are the inherent properties of the polymer, such as its molecular weight, crystallinity, tacticity, and the presence of functional groups, additives, and plasticizers. The type of microorganism and the nature of any pretreatment also play crucial roles. Together, these factors influence the polymer’s susceptibility to enzymatic attack and, consequently, the overall rate and extent of its biological degradation. Environmental factors, such as temperature, humidity, and UV exposure, also significantly impact the rate of polymer biodegradation. Pretreatment methods, like mechanical grinding or chemical modifications, can increase the polymer’s surface area, making it more accessible to microbial enzymes. Enhancing the compatibility of additives and plasticizers with microbial metabolism further improves the overall biodegradation process efficiency [95,96].

Polymer degradation refers to any chemical, physical, or biochemical reaction that involves breaking covalent bonds in the backbone of the polymer, resulting in an irreversible change in its properties due to alterations in the chemical structure and the reduction in molecular weight. The breaking of primary chemical bonds in the main or side chain generates reactive species (free radicals) that are responsible for propagating the degradation process of the polymeric artifact. The initiation of the polymer degradation process is catalyzed by abiotic factors, e.g., heat, light, radiation, humidity, pH of the medium, mechanical stress, and chemical attack. These forms of initiation require activation energy for breaking chemical bonds in the polymer, with the binding energy varying according to the atoms’ connection. Generally, the types of bonds in organic polymers are covalent and usually involve short distances and high energies (1.5 Å and 100 K/mol) [96].

In terms of polymer morphology, enzymatic degradation occurs more easily in the amorphous regions of polymers, as the molecular arrangement in these areas is less dense than in crystalline regions, making them more susceptible to degradation. Additionally, the melting temperature *T_m_* of polymers plays a significant role in enzymatic degradation; as the melting point increases, the rate of biodegradation typically decreases [97,98].

*T_m_* = Δ*H*/Δ*S* (4)

Here, Δ*H* represents the change in enthalpy during the melting process, while ΔS denotes the corresponding change in entropy [98].

Chain scission or bond breaking occurs when the localized energy in a chemical bond exceeds the bond’s energy. When a more unstable bond is positioned inside groups or short branches, its breakage leads to either (i) the loss of that side group or (ii) its modification by inserting new atoms (e.g., oxygen), resulting in polymer degradation. This type of degradation can occur both in the solid and molten states. The energy required for bond scission can be provided in various ways, including heat (thermolysis), water (hydrolysis), oxygen (oxidation), chemistry (solvolysis), light (photolysis), gamma radiation (radiolysis), or mechanical and environmental weathering processes [99].

Hydrolytic enzymes play a pivotal role in biodegradation. Polymers that possess both hydrophobic and hydrophilic segments tend to degrade more readily compared to those containing only one of these structures. Two main types of enzymes, extracellular and intracellular depolymerases, are involved in the biodegradation of polymers. Exoenzymes secreted by microorganisms break down large, complex polymers into smaller molecules, such as oligomers, dimers, and monomers, small enough to penetrate bacterial membranes. These molecules are then used as carbon and energy sources, a process known as depolymerization. Under aerobic conditions, microorganisms primarily produce microbial biomass, CO_2_, and H_2_O as end products, whereas anaerobic processes yield microbial biomass, CO_2_, CH_4_, and H_2_O. Complete degradation of polymer substrates is rarely achieved, as a fraction of the polymer becomes incorporated into microbial biomass, humus, or other natural compounds [100].

#### 4.4.1. Hydrolytic Degradation Mechanisms in Synthetic Polymers

**Figure 17:**
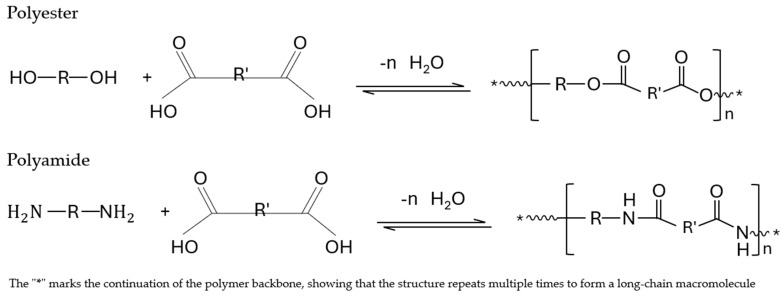
Hydrolytic depolymerization of polyester and polyamide.

Hydrolysis (Figure 17) is a chemical reaction where a bond is disrupted by the interaction with water molecules. It plays a crucial role in the initial stages of synthetic polymer biodegradation, particularly in polyesters. The speed of hydrolytic breakdown depends on factors such as crystallinity, functional group type, molecular weight, polymer structure, morphology, temperature, and pH levels. Lyu and Untereker (2009) [101] outline hydrolytic degradation into three stages: a molecular-level reaction driven solely by chemical reactivity, a second molecular interaction dependent on polymer mobility and water interactions, and a macroscopic degradation driven by water diffusion and erosion.

Hydrolysis can result in two main types of polymer degradation: surface erosion and bulk erosion. In surface erosion, the outer layers of the polymer degrade first, while inner regions remain intact until later stages. In bulk erosion, water penetrates the amorphous regions rapidly, leading to a swift loss of material strength and integrity. Polymers that attract water, or those with water-reactive groups in their backbone, are more susceptible to hydrolysis. During this process, polymers break into two components, which distinguishes true hydrolysis reactions (hydro = water, lysis = breakdown). In non-ionized cases, one fragment gains a hydrogen ion (H+), while the other incorporates a hydroxide ion (OH-).

*CE* + *H*_2_*O* → *COH* + *HE* (5)

The degradation rate of polymers through hydrolysis also depends on their polarity and crystallinity. Hydrophobic polymers degrade more slowly because their reduced water permeability prevents effective interaction with water molecules. Additionally, higher crystallinity in polymers restricts water penetration due to steric effects and strong intermolecular forces, which inhibit plasticization in these ordered regions [102].

#### 4.4.2. Thermolysis and Thermal Decomposition of Polymers

Thermal decomposition, or thermolysis, is a process in which a material breaks down into two or more distinct substances when exposed to heat. For polymers, this reaction occurs independently of external agents like oxygen, forming new compounds distinct from the original material. Thermal decomposition is typically an endothermic reaction, requiring energy to break molecular bonds. However, if the energy of reactants surpasses that of the resulting compounds, the reaction becomes exothermic (Δ*H*), releasing heat and potentially resulting in vigorous chemical interactions or explosions.

A critical distinction often overlooked in thermolysis studies, particularly in thermogravimetric analysis (TGA), is the terminology surrounding “decomposition temperature” versus “degradation temperature”. While decomposition temperature specifically refers to the temperature at which a material breaks into new substances, degradation temperature describes the point where a material experiences a loss in its functional properties, such as changes in color, transparency, antimicrobial activity, or mechanical performance [89].

#### 4.4.3. Chemical Decomposition: Oxidation and Thermo-Oxidative Processes

Decomposition in an oxygen-rich environment not only results in the breaking of σ bonds (R-C-C-R) but also disrupts π bonds (R-C=C-R), leading to the incorporation of an oxygen atom through oxidation. Consequently, oxidation reactions may not always show a reduction in the polymer’s average molar mass but can significantly alter its physical and chemical characteristics, such as color changes. While polymers will degrade under sufficient heat regardless of atmospheric composition, thermal oxidation differs by promoting oxidation reactions that typically decompose materials at lower temperatures compared to thermal decomposition [89].

Thermo-oxidative fission is a self-initiating, multi-stage process consisting of initiation, propagation, and termination. Oxygen is highly reactive and interacts rapidly with free radicals present in the environment. In the initiation step, degradation begins through the formation of radicals (R*) via hydrogen abstraction or the breaking of C-C bonds. During propagation, a free radical (R·) reacts with an oxygen molecule (O_2_), forming a peroxy radical (ROO·). This peroxy radical then abstracts a hydrogen atom from another polymer chain, creating a hydroperoxide (ROOH). Since hydroperoxides are unstable, they break into two radicals, (RO·) and (·OH), which further attack the polymer chains, introducing new radicals and breaking bonds by extracting weak hydrogen atoms [103].

Moreover, the interactions between oxygen and polymer chains accelerate the formation of reactive intermediates, which can further drive the breakdown process. This ensures that structural integrity is compromised through bond fragmentation and radical interactions.

#### 4.4.4. Mechanisms of Polymer Degradation: Chemical and Microbial Interactions

The degradation of polymers is influenced by their inherent characteristics and external factors, such as the presence and diversity of microorganisms, which can vary geographically. Degradation processes are generally categorized as abiotic, involving factors like heat, radiation, chemicals, and moisture, or biotic, involving bacteria, fungi, and algae. Abiotic degradation often marks the initial phase after plastic’s end-of-life, leading to physical and chemical changes without biological interaction, such as alterations in color, cracks, and dimensional changes. Some modifications require advanced analysis, like assessing crystallinity or molecular weight distribution [89].

In natural environments, biotic and abiotic interactions can collaborate to break down organic matter. Certain microbes produce enzymes that directly interact with plastics, bypassing the need for preliminary fragmentation. An example is the breakdown of polyhydroxybutyrate (PHB) facilitated by bacterial and fungal depolymerases. Abiotic factors, meanwhile, can fragment polymers, making these fragments accessible for microbial enzymatic degradation. Typically, degradation begins at the polymer surface, as it is the most exposed to both chemical and biological attacks [99].

#### 4.4.5. Phases of Polymer Biodegradation

Biodegradation occurs in four stages: (bio)deterioration, biofragmentation, assimilation, and mineralization. In (bio)deterioration, microorganisms and abiotic factors break down large materials into smaller fragments. Biofragmentation follows, where enzymes reduce polymers into dimers and monomers. In assimilation, microorganisms integrate these small molecules into their metabolism for energy and biomass. Mineralization simultaneously transforms organic material into inorganic minerals absorbed by the environment and microbes. The efficiency of these stages depends on polymer properties, microbial activity, and environmental conditions. Factors such as temperature, moisture, and oxygen availability significantly impact the degradation rate. Additionally, the presence of additives and plasticizers can either enhance or hinder microbial activity. Understanding these interactions is crucial for improving plastic biodegradation technologies and mitigating environmental impacts [89].

## 5. Quarterly Report—Q1/2024

**Figure 18:**
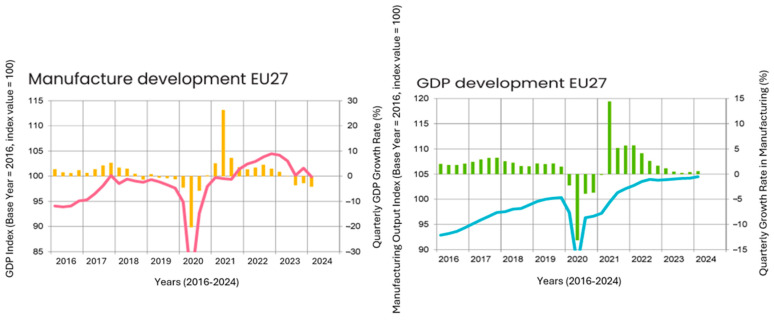
Production index—Q1 2024 [104].

The global economic situation showed only modest improvement compared to the previous quarter. Emerging markets, such as China, exhibited more robust growth relative to developed regions, including the EU27 (Figure 18). The GDP of the EU27 saw a minimal rise of just 0.3% from the previous quarter. Persistent high inflation rates played a significant role in limiting the pace of recovery. In response, numerous central banks raised interest rates to curb inflation, which, while reducing inflationary pressures, also made access to credit more challenging, particularly for businesses seeking to expand. This, in turn, reduced the likelihood of corporate investments [104].

Additionally, geopolitical tensions and protectionist policies have impacted global markets. However, rising wages contributed positively to consumer spending, and global industrial production increased by 1.1% quarter-over-quarter, surpassing the previous year’s figures by 2.8%. This growth mainly stemmed from countries like China, India, and Brazil. On the other hand, production saw declines in the US (−0.2%) and EU27 (−1.7%) compared to the last quarter (Table 3). While Germany and Spain experienced growth, countries like France, Italy, and Poland saw production decreases. Despite high inflation and rising production costs, there remain substantial opportunities to scale up chemical recycling technologies, which can lower material expenses, strengthen supply chain resilience, and support circular economy initiatives. Furthermore, adopting these technologies can enhance long-term sustainability, reduce dependence on virgin materials, and contribute to a more resilient and eco-friendlier industrial ecosystem [104,105].

### 5.1. Automotive Sector

**Figure 19:**
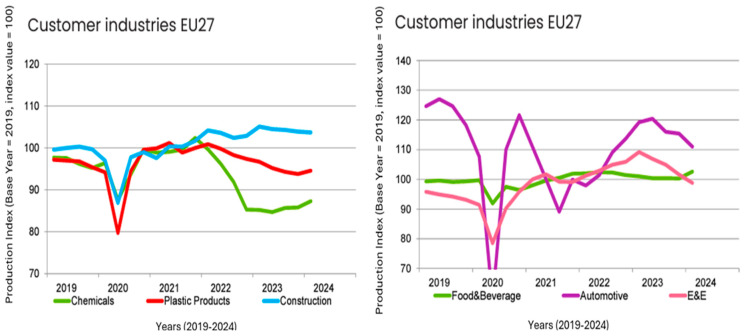
Industrial production of plastics—Q1 2024 [104].

Industrial production in EU27 (Figure 19) contracted by 1.7% in comparison to the previous quarter, impacting key customer industries of plastics manufacturers. The European automotive sector experienced a production decline of 3.8%, marking the third consecutive quarter of negative growth. Demand for electric vehicles in particular remained subdued across Europe. Additionally, the electrical and electronics (E&E) sector saw a downturn in production. The easing of the exceptional conditions brought about by the COVID-19 pandemic, including social distancing measures and the shift to remote work, has resulted in reduced demand for E&E products. The construction sector faced only a slight decrease, although high interest rates continued to exert pressure on activity. This contraction across multiple industries underscores the challenging economic environment for plastics manufacturers reliant on these sectors [104].

While the production of chemical and plastic products saw some recovery, chemical production remained significantly below pre-war levels. Energy-intensive sectors continue to struggle in Europe, facing ongoing challenges. After an extended period of stagnation, the food and beverage industry experienced a rebound in production (Table 4.) [104].

This industrial contraction highlights the vulnerability of plastics manufacturers to broader economic trends, such as shifting consumer demand, supply chain disruptions, and geopolitical uncertainties. Additionally, it emphasizes the need for strategic investments in sustainable manufacturing technologies, including chemical recycling, to improve material efficiency and reduce dependence on volatile markets. Embracing such initiatives can enhance resilience and competitiveness, ensuring long-term sustainability in a rapidly evolving economic landscape [104,105].

### 5.2. Production of Plastics

**Figure 20:**
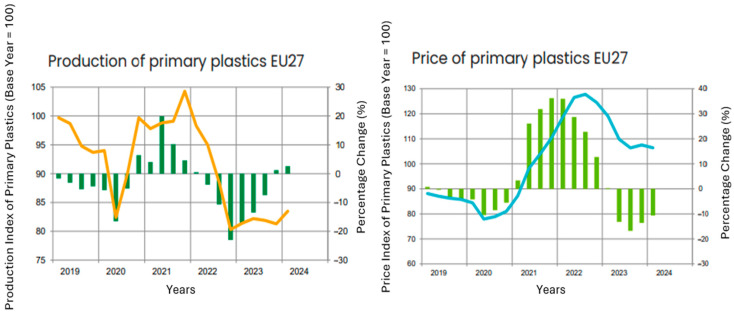
Production of primary plastics—Q1 2024 [104].

The production of plastics in primary forms experienced a notable reduction of 9.7% in 2023. However, this downward trend appeared to stabilize as the new year commenced. During the first quarter of 2024, production demonstrated a 2.7% increase compared to the previous quarter, surpassing levels recorded during the same period in the prior year (Figure 20). This improvement can largely be attributed to growing demand from industries outside Europe, driven by an upswing in global industrial output. Conversely, industrial activity within Europe continued to decline. Nonetheless, European industries slightly raised their orders for plastics, motivated by concerns over potential supply chain disruptions and inadequate inventory levels. Despite these early signs of recovery in 2024, the overall production levels of plastics in primary forms remained subdued. Persistent challenges, including elevated production costs and excessive bureaucratic hurdles within Europe, continued to constrain output [104].

Producer prices for plastics in primary forms experienced a steep decline in 2023, primarily due to a global decrease in demand. Although this trend persisted into 2024, the rate of price reduction slowed. While demand for plastics began to recover, prices remained 1% lower than in the previous quarter and were significantly reduced compared to the same period last year (Table 5.). European manufacturers faced additional challenges due to higher energy costs relative to other regions, which further inflated production expenses. This disparity intensified financial pressures on European producers, further squeezing profit margins. Moreover, the heightened energy costs are exacerbated by stricter environmental regulations, which necessitate significant investments in emissions-reducing technologies. While such measures align with long-term sustainability goals, they add immediate financial strain on producers operating within the European market. Additionally, limited access to affordable feedstocks and reliance on imports contribute to heightened material costs, further reducing price competitiveness on a global scale [104,105].

### 5.3. Foreign Trade

**Figure 21:**
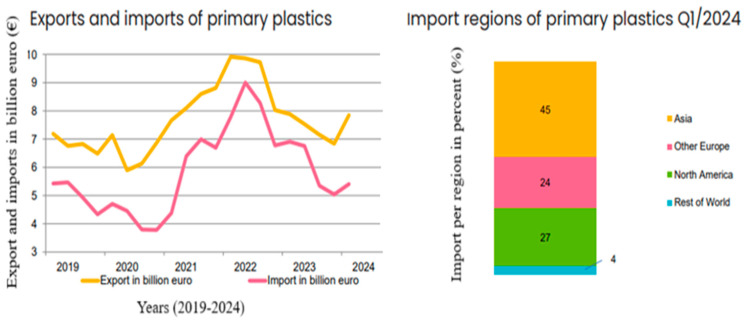
Export and imports—Q1 2024 [104].

In the first quarter of 2024, the export value of plastics in primary forms reached 7.85 billion euros, marking a significant rise compared to the previous quarter (Figure 21). This increase was the first since Q1 2022, driven by a rebound in global economic activity, which boosted international trade. Exports increased across all regions, with the largest gains observed in Europe (excluding the EU) and North America. However, despite the quarterly growth, exports of plastics in primary forms remained lower compared to the same period last year. This rebound in export activity reflects the partial stabilization of global supply chains and a gradual recovery from trade disruptions observed during the previous economic downturns. The growth in demand outside the EU highlights the increasingly interconnected nature of global markets and underscores the importance of fostering trade agreements and improving logistical frameworks to sustain this momentum. Furthermore, the strategic role of plastics in primary forms as key inputs for various industrial sectors, including automotive and construction, suggests potential for further export growth as international production activities normalize [104].

Similarly, the value of imports of plastics in primary forms to the EU27 rose to 5.41 billion euros, with import growth noted across all regions (Table 6.). As with exports, Europe and North America posted the highest growth rates. Despite these increases, imports of plastics in primary forms remained below last year’s levels. The downturn in imports appears to have passed for the EU, signaling the start of recovery. The trade balance improved and stayed positive. The rise in import values reflects both a stabilization of domestic consumption patterns and a strategic effort by EU27 industries to mitigate risks associated with supply chain disruptions. By diversifying sources of imports, industries are aiming to secure consistent access to raw materials essential for manufacturing processes [104,105].

### 5.4. European Plastics Manufacturers

The production of plastics in primary forms rose by 2.7% compared to the previous quarter and exceeded the levels observed in the same period last year. This growth was primarily driven by demand from industries outside of Europe. While global industrial output saw an increase, the EU27 experienced a 1.7% decline in production. Despite signs of recovery, the outlook for European plastics manufacturers remains challenging. Current production levels are still approximately 20% lower than those recorded before the onset of the conflict in Ukraine. High production costs in Europe, compared to other regions, continue to undermine the competitiveness of the continent as a manufacturing base. Additionally, many companies are unable to fully pass on these elevated costs, putting significant pressure on profit margins within the European plastics industry. The ongoing shortage of orders is also a major obstacle for the sector [104].

Moreover, regulatory pressures and sustainability goals further complicate the recovery for European plastics manufacturers. Many companies face increased scrutiny regarding environmental compliance and the transition toward circular economy practices, which often requires substantial investments in green technologies. Combined with high energy costs and logistical challenges, this puts additional strain on production capabilities. Looking forward, the global economic outlook appears to be improving. Inflation rates are declining, and several central banks, particularly in emerging markets, have begun to reduce interest rates. A similar trend may emerge in the Eurozone shortly. Lower inflation and interest rates are expected to stimulate consumer spending and investment, leading to increased demand for industrial goods and plastics. Additionally, the stabilization of global supply chains and easing of geopolitical tensions could positively impact trade and industrial activities [105].

**Figure 22:**
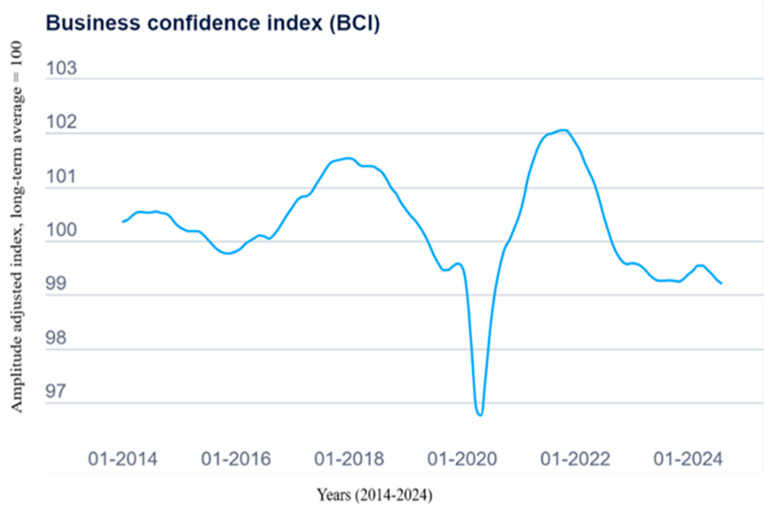
Business Confidence Index (BCI) [105].

The Business Confidence Indicator (BCI), published by the OECD, serves as a tool to track industrial output growth and forecast key turning points in economic activity (Figure 22). This index is based on surveys assessing trends in production, orders, and inventories of finished goods in the industrial sector. A value above 100 indicates optimism regarding near-term business performance, while values below 100 reflect pessimism. In June 2024, the BCI continued its decline, falling to 99.42 points, indicating a cautious outlook. The decline reflects ongoing uncertainty fueled by high inflation rates, geopolitical tensions, and sluggish industrial recovery in key global markets. Despite this, pockets of growth in emerging markets and improving trade conditions offer some positive signals for future business sentiment [105].

## 6. Conclusions and Future Directions

The state of polymer recycling is currently at a critical juncture, as it confronts the escalating environmental challenges associated with plastic waste. Traditional mechanical recycling methods have proven inadequate, prompting a shift towards innovative chemical and biological recycling processes such as pyrolysis, depolymerization, and enzyme-based degradation. These advanced technologies provide more sustainable solutions aligned with the principles of a circular economy, wherein materials are perpetually reused to minimize waste generation. Nevertheless, substantial obstacles persist in scaling these methodologies, including the need to ensure that recycled polymers meet established performance standards.

To advance polymer recycling, future efforts must concentrate on enhancing the scalability and efficiency of these chemical and biological processes. While technologies such as pyrolysis and enzymatic degradation show significant promise, their widespread implementation hinges on the successful navigation of technical challenges and regulatory barriers. The establishment of closed-loop recycling systems is crucial, as they guarantee that materials can be reused without significant degradation in quality.

Furthermore, research initiatives should prioritize the development of recycled polymers that exhibit properties comparable to those of virgin plastics, thereby expanding their applicability across various industrial sectors. Collaboration between industry stakeholders and policymakers will be essential for establishing more robust waste management frameworks, particularly in regions with underdeveloped post-consumer waste infrastructures. The integration of automation, AI-driven sorting technologies, and sustainable product design principles will also play pivotal roles in enhancing recycling initiatives.

The economic context, as highlighted in the Quarterly Report 2024, further complicates the landscape of recycling. The EU27 experienced only minimal GDP growth of 0.3% and a notable 1.7% decline in industrial production, particularly in critical sectors such as automotive and electronics. The automotive industry, which significantly influences plastics demand, contracted by 3.8%, marking its third consecutive quarter of negative growth. Despite these challenges, the production of plastics in primary forms showed early signs of recovery in Q1 2024, with a 2.7% quarterly increase, driven by demand from outside Europe and improved trade dynamics. Producer prices for plastics, although still lower than in the previous year, began to stabilize, signaling a shift toward economic recovery. Additionally, global trade in plastics exhibited resilience, with exports rising significantly compared to the prior quarter, particularly to non-European regions. Imports to the EU27 also increased, reflecting recovering demand and improved trade balances. These trends underscore the importance of addressing cost disparities and fostering innovation to sustain growth in the plastics sector.

In conclusion, while the polymer recycling sector faces formidable challenges, ongoing technological advancements, economic recovery, and collaborative efforts across various industries present a viable pathway forward. By addressing both the technical and economic barriers to effective recycling and aligning with circular economy principles, the sector has the potential to significantly contribute to a more sustainable framework for plastic waste management. This transition, supported by conducive regulatory environments, industrial innovation, and improving market conditions, will be essential in ensuring long-term sustainability amidst evolving environmental and economic pressures.

## Figures and Tables

**Table 1:** Physical and mechanical characteristics of primary plastic materials 1/2 [45].

Material	Morphology	Density (g.cm^−3^)	Glass Transition Temperature (°C)	Melting Temperature (°C)
LPDE	Semi-crystalline (40–55%)	0.91–0.93	−130 to −100/−30 to −10	110–115
HDPE	Semi-crystalline (60–80%)	0.94–0.96	−130 to −100	125–135
PP	Semi-crystalline	0.90–0.91	−20 to −21	160–165
PS	Amorphous or Semicrystalline	1.05	80–105	-
PET	Semi-crystalline	1.33–1.4	70–85	245–260
PVC	Amorphous	1.6–1.35	−50 to −80	-

**Table 2:** Physical and mechanical characteristics of primary plastic materials 2/2 [45].

Material	Initial Degradation Temp. (°C)	Tensile Strength (MPa)	Tensile Modulus (MPa)	Strain at Break (%)
LPDE	487–498	8–23	200–500	300–1000
HDPE	480–498	18–35	700–1400	100–1000
PP	450–470	21–37	1100–1300	20–800
PS	415–425	45–65	3200–3250	3–4
PET	425–445	47	3100	50–300
PVC	290–315	10–25	-	170–400

**Table 3:** GDP and manufacturing development in EU27 [104].

	2023	Q1/24		Q1/24-Q1/24
	% to Year	% to Year	% to Quarter	% to Year
GDP world	2.5	2.4	0.6	2.4
GDP EU27	0.5	0.6	0.3	0.6
Man. World	2.0	2.8	1.1	2.8
Man. EU27	−1.1	−4.1	−1.7	−4.1

**Table 4:** Trends in customer industries in EU27 [104].

	2023	Q1/24		Q1/24-Q1/24
Industry	% to Year	% to Year	% to Quarter	% to Year
Food	−1.5	1.6	2.3	1.6
Automotive	11.6	−6.9	−3.8	−6.9
E&E	−8.1	−9.5	−2.7	−9.5
Plastic	−4.1	−2.2	0.9	−2.2
Chemicals	−8.5	2.4	1.8	2.4
Construction	1.1	−1.3	−0.3	−1.3

**Table 5:** Production and price trends of plastics in primary forms in EU27 [104].

	2023	Q1/24		Q1/24-Q1/24
Industry	% to Year	% to Year	% to Quarter	% to Year
Production	−9.7	2.6	2.7	2.6
Producer prices	−11.0	−10.6	−1.0	−10.6

**Table 6:** Comparison of exports and imports of plastics in primary forms in EU27 [104].

	2023	Q1/24		Q1/24-Q1/24
Regions	% to Year	% to Year	% to Quarter	% to Year
Extra EU27	−0.5	14.7	−21.7	7.3
Asia	7.4	9.7	−27.4	1.2
Rest Europe	−5.9	18.2	−18.0	11.4
N. America	−0.4	20.8	−13.5	17.5

## Data Availability

Not applicable.
